# Reference values for static posturography of sportive and healthy adults aged 18–30 years

**DOI:** 10.1186/s13102-025-01128-z

**Published:** 2025-04-25

**Authors:** Stephan Becker, Anna Thomas, Lilli Ulrich, Laura Becker, Carlo Dindorf, Joshua Berger, Michael Fröhlich

**Affiliations:** 1 Department of Sport Science, RPTU Kaiserslautern-Landau, Kaiserslautern, 67663 Germany; 2 Department of Applied Training Science, German University for Prevention and Health Management, Saarbrücken, 66123 Germany

**Keywords:** Balance, Center of pressure, Sway path length, Area of ellipse, Postural sway, Sensorimotor control, Proprioception

## Abstract

**Background:**

Measuring postural sway and comparing results in a pre-post design or with reference values can increase diagnostic accuracy and the quality of certain treatments and rehabilitation. The process of continuously regulating and sustaining a stable posture involves intricate sensorimotor control.

**Methods:**

All 173 participants (78♂, 95♀; 21.9 ± 2.7 years, 172.6 ± 9.8 cm; 70.0 ± 13.3 kg; BMI: 22.7 ± 2.8) were students in sport and health sciences. Static posturography (open eyes, closed eyes) was performed for 30 s under standardized conditions on a force plate and the parameters analyzed were the sway path length (SPL) and the area of ellipse (AoE).

**Results:**

On average, with eyes open the SPL was 250 ± 116 mm, and the AoE was 125 ± 82 mm^2^ measured with eyes open. With eyes closed, the SPL increased to 337 ± 151 mm and the AoE increased to 175 ± 125 mm^2^. An examination of possible sex differences in static balance revealed only a significant difference for the AoE with closed eyes. In general women achieved slightly better scores than men did. Body height did not have an influence on balance.

**Conclusion:**

The present dataset provides reference values for young and sportive adults between 18 and 30 years of age. An examination of possible gender differences in static balance revealed a significant difference only between men and women for AoE with closed eyes. Body height does not have an influence on balance but the literature is inconsistent and further research is needed. Owing to the popular use of posturography in practice, further reference values are needed, and more attention should be given to ΔSPL and ΔAoE (open eyes, closed eyes) as well as the interpretation of the relationship between the SPL and AoE.

## Introduction

Static balance, defined as the ability to maintain a stable and upright posture while remaining stationary, is a fundamental aspect of human physiology and biomechanics. Static balance is pivotal for performing everyday activities safely and effectively, ranging from standing still to executing complex movements. There is a close connection between static balance and sensorimotor control [1]. Sensorimotor function involves the integration of sensory inputs (e.g. proprioception, vision, and vestibular input) with motor outputs to control movements and maintain body position in space [2, 3]. Various receptors in the body, including proprioceptors (muscle spindles, Golgi apparatus), exteroceptors (Vater-Pachini corpuscles, Merkel cells, and Ruffini receptors) in muscles, joints, and skin, visual receptors in the eyes and vestibular receptors in the inner ear, provide information about body position, orientation, and movement [1, 3]. Poor static balance may be due to poor sensorimotor status, which can be improved through training [4], if a person’s individual background of the person (e.g.: age, gait abnormalities, injury) deems it necessary..

Static balance is essential across all age groups, as it helps prevent falls and related injuries, enhances athletic performance, and supports general mobility. In children, the development of balance is crucial for motor skills and physical activity. For adults, good balance supports the execution of daily tasks. In older populations, maintaining static balance is critical for preserving independence, preventing falls, and enhancing quality of life [5, 6]. Therefore, evaluating and understanding static balance is of paramount importance for promoting health and well-being across the lifespan.

There are several proven methods for measuring balance, with computer-assisted posturography, also known as postural stability test, being one of the most established static procedures [7, 8]. Fluctuations in the center of mass (COM) can be measured via the center of pressure (COP) with the help of a force plate, which also occurs in healthy people at an absolute standstill and can be measured by the postural parameters, including sway path length (SPL) and area of ellipse (AoE). Table 1 shows the results of a few studies that represent comparative data for posturographic measurements, whereby only [4] is actually suitable for a direct comparison with our sample owing to the age of the participants.

**Table 1:** Overview of similar posturographic investigations with eyes open and eyes closed

**Author**	**N**		**Age** **[years]**	**Time** **[s]**	**SPL** **[mm]**		**Δ** **[%]**	**AoE** **[mm**^**2**^**]**		**Δ** **[%]**
	**Groups**				**OE**	**CE**		**OE**	**CE**	
[4]	male	47	21 ± 2	30	326 ± 82	380 ± 102	17	95 ± 60	127 ± 101	34
	female	55	21 ± 2		345 ± 70	395 ± 84	14	96 ± 68	113 ± 68	18
[9]	young	10	29 ± 5	30	273 ± 48	404 ± 121	48	223 ± 129	326 ± 210	68
	old	10	78 ± 4		364 ± 163	647 ± 469	78	173 ± 93	305 ± 250	76
[10]	young	20	23 ± 2	20	99 ± 14	110 ± 16	11	-	-	
	old	20	68 ± 4		111 ± 15	134 ± 16	21	-	-	
[11]	-	174	21-69	30	485 ± 215	-		70 ± 44	-	

SPL = sway path length and AoE = area of ellipse (mean ± standard deviation); different groups (sex, age); measurement time (20 s, 30s); OE = open eyes and CE = closed eyes; Δ describes the percentage difference between OE and CE

A frequently observed problem with individual balance ability (static and dynamic) is the dominance of the visual sense [4, 12, 13]. Without the feedback of visual receptors into the sensorimotor control circuit, most people find it much more difficult to achieve comparable values in a balance measurement [14]. If the difference exceeds a certain level, it might indicate poor sensorimotor status, as the afferent signals from the proprioceptors, mechanoceptors and the vestibular system are not adequately processed to maintain balance in the best possible way and lead to a fast and effective motor output [1, 11]. For this reason, posturography is usually performed with open eyes (OE) and closed eyes (CE) (see Table 1).

With respect to sex-specific differences in postural sway PW Overstall, AL Johnson and AN Exton-Smith [15] examined 505 test subjects considering postural sway. According to their results, sex has a significant effect on postural sway. JW Kim, GM Eom, CS Kim, DH Kim, JH Lee, BK Park and J Hong [16] investigated sex-specific differences, as being older and female are statistically associated with a greater risk of falling [17, 18]. Among the older test participants, the women had poorer values [16]. A Lipowicz, M Bugdol, K Graja, K Nowakowska-Lipiec, K Jochymczyk-Woźniak, D Fryc, R Michnik and A Mitas [4] reported in generally higher values (AoE, mean velocity COP) for women in a younger sample (20.7 ± 1.9 years). However, other studies did not reported sex-specific differences [10, 11, 14, 19, 20].

Body height might affect postural sway [21–23]. Research has shown that individuals with greater body height, including those with greater body mass or greater stature, may experience differences in postural control and sway [22, 24]. Similarly, an increased or decreased COM affects the postural sway [25]. Additionally, muscle mass and strength, which can vary with body height, also play crucial roles in maintaining balance and stability [17, 24]. Therefore, it is conceivable that changes in body height might impact how the body adjusts to maintain balance, leading to variations in postural sway.

Reference values offer the possibility of interpreting measurement results if there are no comparable intraindividual values (side-by-side comparison, pre-post comparison). These values are only a guide, and their importance must always be judged upon a case-by-case basis; however, they are very helpful in practice, since they serve as an orientation to measurement interpretation, especially for people with less experience, and they allow for a deeper interpretation when working with cross-sectional or longitudinal data. As the data availability is particularly lagging for young adults (see Table 1), with only one substantial study [4] analyzing 102 young adults between the ages of 20 and 24 years with a comparable sporting background. These study data are used with respect to the following points:

- 1^st^ aim: A descriptive overview of the reference values of young and sportive adults between 18 and 30 years of age during static posturography.
- 2^nd^ aim: Data used to check potential sex differences.
- 3^rd^ aim: Data used to determine whether body height affects the postural sway during posturography.

The literature shows that there are relatively few reference values for this type of balance measurement. The dataset provides new reference values (young adults, 18–30 years) with a comparatively large and homogeneous sample. These reverence values can be used for comparable people, for example when testing balance in preventive settings, to help interpreting the measurements. Equally, the data can represent a target value for injured persons as a part of their rehabilitation program.

## Materials and methods

### Subjects

The study was based on and carried out in accordance with the current guidelines of the Declaration of Helsinki and was approved by the institutional ethics commission RPTU Kaiserslautern-Landau (No. 55). All participants signed informed consent forms and provided permission to publish the results. The authors have no conflicts of interest to declare.

All 173 participants (78♂, 95♀; 21.9 ± 2.7 years, 172.6 ± 9.8 cm; 70.0 ± 13.3 kg) were students of sport and health sciences (between 18 and 30 years of age), with an overall weekly sports activity duration of 120 to 240 min. All participants had normal vision or corrected-to-normal vision with glasses. The exclusion criteria were an age > 30 years, a history of balance deficits, neurological disorders, the use of medication or drugs that could affect balance, illness, and acute complaints or injuries.

### Test procedure

Static posturography was performed in a laboratory under standardized conditions. After an explanation of the content and procedure of the study (standardized written and verbal instructions), personal data were recorded. The chronology of the posturographic measurements (open eyes, closed eyes) was randomized for each individual. The force plate was recalibrated before each measurement. The participants positioned themselves in a hip-width stance in the neutral-zero position on the force plate and were barefooted. Depending on the randomization result, for both test conditions, data were recorded for 30 s each with a break of one minute between the two tests (OE, CE). All participants were given the clear instruction (written and verbal) to stand as still as possible and to visually focus a wall marker (distance: 2.0 m). The SPL and the AoE were determined for each measurement (OE, CE).

### Static posturography

The measurements were carried out with a force plate (FDM 1.5, Zebris Medical, Germany) using MR3 software (version *3.20.60*, Noraxon, USA). The measurement range of the plate is 1–120 N/cm^2^, and the measurement rate was 120 Hz with 11264 sensors (64 × 176) [26].

#### Sway path length (SPL)

The sway path length describes the sway of the center of pressure (COP), which is proportional to the horizontal acceleration of the center of mass (COM). These movements are also known as postural sway. This parameter describes the movements of the COP as a distance (mm or cm). The following terms for the same parameter can be found in the literature: center of pressure displacement, center of pressure track, and center of pressure path.

#### Area of ellipse (AoE)

The area of ellipse describes the horizontal movements of the COP as an area (mm^2^ or cm^2^). These movements also belong to the category of postural sway. The following terms for the same parameter can be found in the literature: sway area, area of postural balance, center of pressure area, confidence ellipse area, and displacement area.

### Statistics

All the statistics were calculated with IBM SPSS (29.0 for Macintosh, Chicago, IL, USA). The results are presented as mean values ± standard deviations as well as median and interquartile ranges because of the nonnormal distribution of the data.

To remove outliers from the dataset, z-standardization was carried out so that the values (AoE, SPL) were comparable. For this purpose, the mean value of the distribution (μ) was subtracted from each value (X) and divided by the standard deviation of the distribution (σ).

$$Z=\frac{X- \mu }{\sigma }$$

All z values > 3 and less than < − 3 were declared as outliers, and the data were removed from the dataset. This reduced the sample from 184 to 173 people. Reference values were subsequently calculated for these participants on a descriptive basis (1 st aim). The Kruskal–Wallis test was used to analyze the data for sex differences (2nd aim), as the data were not normally distributed. Spearman’s rank correlation coefficient (ρ) was calculated, as the data were not normally distributed, to determine whether body height might influence balance in static posturography (3rd aim). The significance level was set at *p* < 0.025 after a Bonferroni correction, since SPL and AoE were always measured in one trial (OE, CE). Spearman’s rho is provided as an effect size for significant results (0.1 = small effect; 0.3 = medium effect, 0.5 = large effect).

## Results

### Reference values (1^st^ aim)

Table 2 provides an overview of the descriptive data. Between the OE and CE conditions the average difference for **Δ**SPL is 34% and 40% for **Δ**AoE.

**Table 2:** Descriptive data (mean ± standard deviation, median, interquartile range, percentiles) of all four posturographic parameters

Parameter	N	Mean ± SD	Median	ICR	5	10	25	50	75	90	95
SPL OE [mm]	173	250 ± 116	224	147	101	130	163	224	310	417	464
SPL CE [mm]	173	337 ± 151	316	202	147	163	220	316	422	561	650
AoE OE [mm^2^]	173	125 ± 82	105	89	34	46	69	105	158	228	308
AoE CE [mm^2^]	173	175 ± 125	135	125	52	64	92	135	217	366	441

*SPL* sway path length, *AoE* area of ellipse, *OE* open eyes, *CE* closed eyes

### Sex differences (2^nd^ aim)

Table 3 provides a sex-specific overview of the sample comparing 78 ♂ (22.4 ± 2.7 years, 180.3 ± 7.5 cm; 79.6 ± 11.7 kg) and 95 ♀ (21.6 ± 2.7 years, 166.3 ± 6.6 cm; 62.3 ± 8.6 kg) individuals. The Kruskal–Wallis test revealed a significant difference between men and women only for AoE CE (H(1) = 6.725; *p* = 0.01). There were no sex differences in AoE OE (H(1) = 1.137, *p* = 0.29), SPL OE (H(1) = 1.230, *p* = 0.27), or SPL CE (H(1) = 2.203, *p* = 0.14).

**Table 3:** Sex-specific descriptive data (mean ± standard deviation, median, interquartile range) were obtained for all four posturographic parameters

Sex	Parameter	N	Mean ± SD	Median	ICR
♀	SPL OE [mm]	95	242 ± 117	220	131
	SPL CE [mm]	95	322 ± 155	286	200
	AoE OE [mm^2^]	95	128 ± 75	100	87
	AoE CE [mm^2^]	95	156 ± 114	113	127
♂	SPL OE [mm]	78	259 ± 114	229	165
	SPL CE [mm]	78	355 ± 147	328	200
	AoE OE [mm^2^]	78	134 ± 91	116	98
	AoE CE [mm^2^]	78	197 ± 136	151	127

*SPL* sway path length, *AoE* area of ellipse, OE open eyes, *CE* closed eyes

### Correlation between body height and posturography (3^rd^ aim)

No correlation was detected between the persons’ heights and the results of static posturography (see Table 4).

**Table 4:** Spearman correlation coefficient (ρ) for the participants’ heights and all four posturographic parameters

Parameter	N	ρ	Sig
SPL OE	173	0.03	0.72
SPL CE	173	0.11	0.15
AoE OE	173	0.01	0.88
AoE CE	173	0.12	0.11

*SPL* sway path length, *AoE* area of ellipse, *OE* open eyes, *CE* closed eyes

## Discussion

The present dataset provides reference values for young and sportive adults between 18 and 30 years of age, which was the main objective (1st aim) of this study. On average, a SPL of 250 ± 116 mm and an AoE of 125 ± 82 mm^2^ were measured with the eyes open. With eyes closed, the SPL increased to 337 ± 151 mm, and the AoE increased to 175 ± 125 mm^2^. An examination of possible sex differences in static balance revealed a significant difference only for the AoE with CE (2nd aim). In general women achieved slightly better scores than men did. Body height does not seem to have any influence on balance (3rd aim). Further interpretation of the data is provided in the corresponding sections.

### Reference values (1^st^ aim)

Identifying factors that affect balance can increase diagnostic accuracy and the quality of specific treatments and rehabilitation. The process of continuously regulating and sustaining a stable posture in static and dynamic situations involves intricate sensorimotor control. Even in young adults, COM and COP beneath the feet exhibit continuous movement, even when standing still [11]. In particular, the comparison of measurements with OE and CE seems to be important, as the visual sense plays a dominant role (see Tables 1 and 2) within the sensorimotor control loop [4, 12, 13], but in everyday and sporting situations, we do not always visually anticipate or capture everything (e.g., supination trauma) [4]. People who have good sensorimotor feedback without visual sense are able to react more quickly to changes (e.g., joint kinematics) because ofthe sensorimotor control loop between receptors, the central nervous system and effectors [27]. This also includes the relatively small changes in the COM or COP, as shown here in static postugraphy [28]. If one follows Pomarino’s classification [11], values of healthy participants aged between 18 and 30 years that are above the 90 th percentile can already be classified as conspicuous, meaning for those participants that preventive actions to improve balance are advisable for these participants. For the present study, the 90 th percentile corresponds to a SPL > 417 mm and an AoE > 228 mm^2^ with OE and a SPL > 561 mm and an AoE > 366 mm^2^ with CE. The only sample with which our results can be compared is that of A Lipowicz, M Bugdol, K Graja, K Nowakowska-Lipiec, K Jochymczyk-Woźniak, D Fryc, R Michnik and A Mitas [4]. They examined 102 young adults with an average age of 21 years who also had an affinity for sports. On average, the SPL in their sample was slightly greater than that in ours, and the AoE was slightly lower, but from our point of view, it was within a comparable range. The other investigations seem roughly similar, but the samples were too small [9, 10], the measurement duration of 20 s was too short [10], or the age distribution did not allow for comparison [11]. More investigations and larger datasets are needed. In particular, across all age groups in order to better understand the development of the parameters (AoE, SPL) over the lifespan.

Another point that should be given greater attention in future research is the difference between OE and CE conditions. The difference between OE and CE measurements could be useful for better assessing a person's proprioceptive status. If performance with CE decreases too much, it indicates the dominance of the visual sense and the lagging processing of proprioceptive signals in the CNS. The average difference between OE and CE measuremetns in the data of A Lipowicz, M Bugdol, K Graja, K Nowakowska-Lipiec, K Jochymczyk-Woźniak, D Fryc, R Michnik and A Mitas [4] was 16% lower for **Δ**SPL (present study: 34%) and 26% for **Δ**AoE (present study: 40%). The available data (reference values) in the scientific community is still lagging to draw precise limits (conspicuous versus inconspicuous) in the sense of a preventive guidance. However, if the present study is combined with that of A Lipowicz, M Bugdol, K Graja, K Nowakowska-Lipiec, K Jochymczyk-Woźniak, D Fryc, R Michnik and A Mitas [4] and the average is used as a guide for healthy adults in this age range, then difference in **Δ**SPL of 25% (OE versus CE) and difference in **Δ**AoE of 33% (OE versus CE) difference could be considered as alarming signs in the context of a preventive check. One consequence of this could be to increasingly integrate proprioceptive exercises into everyday life or training to improve values on the basis of the identified weaknesses. Finally, how different parameters of postural sway can be compared and interpreted remains to be clarified. The velocity at which the COP fluctuated was not recorded in this study. Typically, a high COP velocity is associated with a balance issue [29, 30]. A low area of the ellipse means that the COP is moving within a more limited space, which could indicate a more stable balance and controlled sway. A possible explanation could be that the central nervous system is able to correct deflections faster and in a smaller area. However, this could also mean that a high COP velocity and a high COP SPL would not necessarily be bad, as long as they take place in a correspondingly small AoE. Consequently, a high COP SPL or a high COP velocity alone are not necessarily indicators of poor balance or a poor proprioceptive status. The area within which the COP moves appears to be much more important for healthy subjects without neurological disorders from our point of view. Further studies to verify these hypotheses with a combination of the aspects mentioned are needed. Other findings indicate that the central nervous system may intentionally produce ground-reaction forces independently of body sway, possibly utilizing sway as an exploratory mechanism to facilitate continuous sensory input [31]. These findings support the approach that more attention should be given to the combined interpretation of the parameters (SPL, AoE), as well as the relationsships (**Δ**SPL,** Δ**AoE) between OE and CE measurements. The physiological mechanisms in this context are too complex to evaluate balance using just one parameter, and not all processes are fully understood [31].

### Sex differences (2^nd^ aim)

The results do not support the hypothesis of a general sex-specific difference with regard to postural sway (SPL, AoE). A significant difference was detected only for AoE EC. For this parameter, men presented a significantly higher values than women did, which is in line with the higher values (non-significant) of men in this sample. A general argument justifying this hypothesis is that women have a statistically greater prevalence of falling [17, 18]. Some studies have also been able to support this by reporting a greater postural sway for women, but primarily by comparing elderly women and men (> 60 years) [5, 32], as well as for young adults (21 years) [4]. No sex differences were reported by D Pomarino, A Nawrath and J Beyer [11], who compared 193 male and 238 female subjects, each of whom were divided into four age groups (2–6 years, 7–10 years, 11–20 years and 21–69 years). F Palazzo, A Nardi, N Lamouchideli, A Caronti, A Alashram, E Padua and G Annino [10] compared the COP parameters between young (n = 20) and older (n = 20) groups with the same sex distribution and reported no sex-specific differences. These results from studies of children, teenagers and young adults do not support this hypothesis [3, 10, 11, 19, 20], and further research is needed. The possible reasons for sex-specific differences between elderly men and women range from differences in muscle mass and strength [33], general differences in body height [36], social factors such as multitasking [34] and general social behavior [35] to psychological factors such as the fear of falling [36], which in turn might lead to less activity and ultimately poorer muscle strength and balance.

### Correlation between body height and posturography (3^rd^aim)

The present sample revealed no correlation between body height and postural sway (SPL, AoE). The hypothesis is based on the idea that taller people have a higher center of mass. The greater the distance between the measuring plate and the COM is, the more artifacts the COP is subject to. Conversely, people with a lower COM should have lower SPL and AoE values. In the literature, several studies have revealed a correlation between body height and postural sway. AJ Lee and WH Lin [37] and P Allard, ML Nault, S Hinse, R LeBlanc and H Labelle [38] reported that people with ectomorphic body types had greater postural sway than people with endomorphic and mesomorphic body types did. A Lipowicz, M Bugdol, K Graja, K Nowakowska-Lipiec, K Jochymczyk-Woźniak, D Fryc, R Michnik and A Mitas [4] analyzed 102 adults of both sexes and reported that the range of maximum sway (AoE) increased with the height and that its COM was statistically significant only for women and not for men. AC Alonso, L Mochizuki, NM Silva Luna, S Ayama, AC Canonica and JM Greve [24] support their results for women and men. They also explained the connection via the increased COM in taller people and the inverted pendulum model, which is based on the relationship between the moving pendulum and its length, mass and stiffness [4]. Nevertheless, the findings with only moderate [39] or slight [23] correlations between body height and balance are less clear. In contrast, G Olchowik, M Tomaszewski, P Olejarz, J Warchoł and M Różańska-Boczula [40] concluded on the basis of their results that shorter people and those with a lower BMIs presented a greater imbalance caused by platform perturbations but with different measurement methodologies. Despite the logic that a greater COP should be associated with greater postural sway, further research is needed. Future studies should include more characteristics, such as the length of the extremities, trunk, etc., which can also have an influence [4].

### Limitations

While the homogeneity of the sample can be seen as an advantage, it also represents a limitation. Many comparative studies have inlcuded heterogeneous groups to better examine age differences. In the present study, it therefore made little sense to investigate the influence of age. The participants were all sportive and healthy, and were studying sport and health sciences, and there were an age difference of only a few years. In particular, the sample characteristic of sportiness limits gerneralizabilty, as it cannot be exlcuded that the values in the present study are better than the general average. The only comparable data [4], which were also obtained from sports students and are therefore comparable, were worse for the SPL values and better for the AoE values in the comparative study. This variance underlines the need for further comparative values. Another limitation is that the speed of the COP movements was not analyzed. In retrospect, this parameter should also be included, as it could be helpful for a general interpretation in combination with the SPL and AoE.

## Conclusions

The present dataset provides reference values for young and sportive adults between 18 and 30 years of age (1st aim). An examination of possible sex differences in static balance revealed only one significant difference between men and women for AoE EC (2nd aim). Body height does not influence balance but the literature is inconsistent, and further research is needed (3rd aim). Owing to the popular use of posturography in practice, further reference values are needed, and more attention should be given to ΔSPL and ΔAoE as well as the interpretation of the relationship between the SPL and AoE.

## Data Availability

The data can be obtained upon request from the corresponding author.

## Abbreviations

AoE: Area of ellipse
CE: Closed eyes
COM: Center of mass
COP: Center of pressure
ICR: Interquartile range
OE: Open eyes
SD: Standard deviation
SPL: Sway path length
ΔAoE: Describes the percentage difference between the areas of ellipses with open eyes and those with closed eyes
ΔSPL: Describes the percentage difference between the sway path lengths with open eyes and those with closed eyes
